# Blue-Light Photobiomodulation in Pediatric Burns: Inflammation, Healing, and Early Scar Outcomes in a Retrospective Historical Cohort

**DOI:** 10.3390/ebj7040050

**Published:** 2026-09-28

**Authors:** Biagio Nicolosi, Emanuele Buccione, Lorenzo Drago, Benedetta Virginia Difalco, Hamilton Dollaku, Michele Rossi, Flavio Facchini, Greta Ghizzardi, Rosario Caruso, Guido Ciprandi

**Affiliations:** 1 Health Professions Department, Meyer Children’s Hospital IRCCS, 50139 Florence, Italy; benedetta.difalco@meyer.it; 2 AISLeC, Italian Nursing Association of Wound Care, 47923 Rimini, Italy; 3 Pediatric Intensive Care Unit, Local Health Authority 3 of Pescara, 65124 Pescara, Italy; emanuele.buccione@asl.pe.it; 4 UOC Laboratory of Clinical Medicine with Specialized Areas, IRCCS MultiMedica, 20138 Milan, Italy; lorenzo.drago@unimi.it; 5 Department of Biomedical Sciences for Health, University of Milan, 20133 Milan, Italy; rosario.caruso@unimi.it; 6 Cardiac Rehabilitation Unit, IRCCS Don Gnocchi Hospital, 50143 Florence, Italy; hdollaku@dongnocchi.it; 7 Unit of Biostatistics, Golgi Cenci Foundation, 20081 Abbiategrasso, Italy; michele.rossi.od@gmail.com; 8 Simple Departmental Organizational Structure of Plastic Surgery and Burn Center, Meyer Children’s Hospital IRCCS, 50139 Florence, Italy; flavio.facchini@meyer.it; 9 Directorate of Nursing and Allied Health Professions, ASST di Lodi, 26900 Lodi, Italy; greta.ghizzardi@asst-lodi.it; 10 Health Professions Research and Evidence Transfer Unit, IRCCS MultiMedica, 20099 Sesto San Giovanni, Italy; 11 Complex Unit of Plastic Surgery, University of Padua, 35128 Padua, Italy; guido.ciprandi@unipd.it

**Keywords:** pediatric burns, photobiomodulation, blue light, wound healing, inflammation, scar, pediatric burn care

## Abstract

**Highlights:**

**What are the main findings?**

- Children treated with adjunctive blue light photobiomodulation showed a greater reduction in local inflammation during the first 72 h after hospital admission than those receiving standard care alone.
- Blue-light photobiomodulation was associated with shorter healing time, better early scar quality, and fewer unplanned dressing changes. The association with autologous skin grafting was not statistically significant.

**What are the implications of the main findings?**

- The observed associations support further investigation of blue light photobiomodulation as an adjunct to standard pediatric burn care.
- These findings provide a rationale for prospective multicenter randomized trials to determine the role of blue light photobiomodulation in routine pediatric burn care.

**Abstract:**

**Background**: Blue-light photobiomodulation (PBM) may support burn wound repair through modulation of early inflammatory responses, but comparative evidence in children remains limited. We examined associations between adjunctive PBM, early local inflammation, wound healing, and early scar quality in pediatric burns. **Methods**: This single-center retrospective historical cohort study included 84 children treated in 2024: 50 historical controls receiving standard care and 34 patients receiving standard care plus blue-light PBM after its introduction into routine practice. The primary outcome was the exploratory composite inflammation score at 72 h, adjusted for the pre-exposure 24 h score and clinical covariates. Secondary outcomes included time to complete re-epithelialization, Vancouver Scar Scale (VSS) score approximately three months after complete re-epithelialization, unplanned dressing changes, and autologous skin grafting. Documented PBM-related adverse events were also reviewed. **Results**: PBM was associated with a lower adjusted 72 h inflammation score (mean difference −2.70 points, 95% CI −3.71 to −1.68), shorter healing time (−5.98 days, 95% CI −8.68 to −3.28), and lower VSS scores (−1.46 points, 95% CI −2.46 to −0.47). The adjusted odds of unplanned dressing changes were lower with PBM (OR 0.25, 95% CI 0.07–0.79), whereas the association with skin grafting was not statistically significant (OR 0.63, 95% CI 0.17–2.16). No PBM-attributable adverse events were documented. **Conclusions**: These hypothesis-generating associations require prospective confirmation and do not establish a causal treatment effect. The sample size and retrospective recording limit conclusions about safety.

## 1. Introduction

Burn injuries in children remain a significant clinical challenge because wound healing must be achieved while preserving skin function and limiting the risk of long-term scarring. In addition to burn depth and extent, the duration of wound closure is particularly relevant in pediatric patients, as delayed re-epithelialization is associated with prolonged inflammation and a greater risk of hypertrophic scar formation [1,2,3]. Early management therefore aims not only to achieve wound closure but also to create a local environment that supports tissue repair while limiting excessive or persistent inflammation.

Inflammation is an essential component of the normal response to thermal injury; however, when prolonged or poorly regulated, it may interfere with keratinocyte migration, re-epithelialization, and subsequent tissue remodeling [4,5]. This relationship is particularly relevant in pediatric burns, where scar development may have functional and aesthetic consequences that persist throughout growth. Strategies capable of modulating the early wound environment without interfering with physiological repair may therefore represent a useful adjunct to established burn-care protocols.

Photobiomodulation (PBM) has been investigated in acute and chronic wound models [6,7,8,9]. Its biological effects depend on wavelength, irradiance, fluence, exposure time, and tissue characteristics. Experimental blue-light studies have reported reactive oxygen species generation in cellular systems [10] and changes in inflammatory-cell responses and tissue organization in animal wounds [11,12,13]. These parameter-dependent findings provide a rationale for research but do not demonstrate a clinical anti-inflammatory mechanism. Blue light at 405 nm has also inactivated bacterial pathogens in laboratory experiments [14]; this finding should be distinguished from evidence of clinical infection control. Clinical wound-healing studies and experimental cellular studies address different questions, and their results should not be treated as interchangeable evidence [8,9].

Despite increasing interest in PBM, the available evidence remains heterogeneous with regard to wound etiology, treatment parameters, and study population. Most experimental evidence on burn healing derives from preclinical models, while clinical studies of blue-light PBM have predominantly involved adult patients or heterogeneous wound populations [9,15,16,17,18,19,20]. Evidence specifically addressing acute pediatric burns remains limited. Preliminary experience in pediatric wound care has suggested that blue-light PBM is feasible and well tolerated in children with skin injuries of different etiologies; however, these observations were based on small case series and did not specifically examine a comparative cohort of children with acute burns [21]. A systematic review by Pradal et al. found no statistically significant improvement in wound contraction or collagen deposition in its meta-analysis of four preclinical studies using 660 nm laser treatment [22]. These findings highlight the uncertainty surrounding PBM in burns. Evidence obtained with red or near-infrared light cannot be directly extrapolated to blue-light devices, given the differences in wavelength and exposure parameters. Blue-light PBM is investigated here as an adjunct to standard burn care; this study does not establish it as a standard treatment for pediatric burns.

This study examined the association between adjunctive blue-light PBM and early local inflammation, time to re-epithelialization, and early scar outcomes in a comparative cohort of children with acute burns. These outcomes were evaluated within the same cohort; the observational design did not allow causal or mechanistic relationships between them to be established.

We hypothesized that adjunctive blue-light PBM would be associated with a lower local inflammatory response at 72 h compared with standard burn care alone. Accordingly, the predefined primary analytical endpoint was the composite local inflammation score at 72 h, adjusted for the corresponding 24 h baseline value. Secondary outcomes included time to complete re-epithelialization, individual inflammatory domains, early scar outcome assessed using the Vancouver Scar Scale approximately three months after complete re-epithelialization, unplanned early dressing changes, and the requirement for autologous skin grafting.

## 2. Materials and Methods

### 2.1. Ethical Approval

The study was conducted in accordance with the principles of the Declaration of Helsinki and with current European and national legislation on the protection of personal data (EU Regulation 2016/679—GDPR and Legislative Decree 196/2003, as amended by Legislative Decree 101/2018). The study protocol was approved by the Pediatric Ethics Committee of the Region of Tuscany (Protocol No. 126/2025, approved on 26 June 2025). As this is a retrospective observational study based on the analysis of clinical data collected during normal clinical practice, the Ethics Committee authorized an exemption from the requirement to obtain informed consent, in accordance with current legislation. Data were collected and analyzed in pseudonymized form to ensure the confidentiality of all participants. Appropriate technical and organizational measures were put in place to protect personal data throughout all stages of the study.

### 2.2. Study Design and Setting

A total of 137 pediatric patients with burn injuries treated at the Pediatric Burns Centre between 1 January and 31 December 2024 were assessed for eligibility. Of these, 53 were excluded: 12 because of a non-thermal burn mechanism, 8 because TBSA was outside the predefined 1–25% range, 22 because the available clinical documentation was insufficient to adequately characterize the burn or treatment course, and 11 because data required to assess the primary outcome were incomplete.

The final study population comprised 84 patients with acute thermal burns. One patient in the historical control group sustained a thermal burn caused by an electrical arc, without current passage through the body. This injury was recorded as “electrical” in the database but was included as a thermal burn. Of these, 50 patients treated between 1 January and 17 July 2024 constituted the historical control group, while 34 patients treated between 18 July and 31 December 2024 constituted the PBM group.

The participant selection process and reasons for exclusion are summarized in the study flow diagram (Figure 1). The study was reported in accordance with the Strengthening the Reporting of Observational Studies in Epidemiology (STROBE) recommendations.

### 2.3. Patient Selection

The study population consisted of pediatric patients aged between 0 and 18 years with a diagnosis of burns who were admitted to the Burns Centre at our Pediatric Hospital between 1 January 2024 and 31 December 2024. The population comprised children and adolescents with burns of varying etiology, extent and depth, managed in accordance with the care protocols adopted by the center during the observation period.

#### 2.3.1. Inclusion Criteria

1. Patients aged between 0 and 18 at the time of admission.
2. Clinical diagnosis of an acute skin burn resulting from hot liquid, contact, flame or another documented thermal cause.
3. Burn area covering between 1% and 25% of the total body surface area (Total Body Surface Area, TBSA).
4. Availability of clinical data relating to the assessment of local inflammation, including erythema, exudate, pain and pruritus, recorded within the timeframes specified in the care protocol.
5. Availability of key clinical outcomes, including healing time, the need for early dressing changes or for skin grafting, and scar assessment using the Vancouver Scar Scale.
6. Availability of comprehensive documentation on the treatment received, including any exposure to blue light photobiomodulation.

#### 2.3.2. Exclusion Criteria

1. Patients with chemical burns, radiation burns, or electrical injuries involving current passage through the body were excluded. Thermal burns caused by an electrical arc without current passage through the body were eligible.
2. Incomplete or missing clinical data relating to the initial characteristics of the burn, including the burning agent, the extent of the lesion or the depth of the burn.
3. Lack of comprehensive information on the course of treatment, including the initial medication, any subsequent treatments, the use of photobiomodulation and any surgical procedures carried out.
4. Lack of clinical assessments of local inflammation (erythema, exudate, pain and pruritus) within the timeframes specified in the care protocol, or lack of key outcome data, including healing time, the need for skin grafting and scar assessment using the Vancouver Scar Scale (VSS).

### 2.4. Exposure Definition and Cohort Allocation

Patients were classified into two groups according to the treatment received during the study period. The PBM group comprised patients treated with blue-light photobiomodulation in addition to the center’s standard burn-care protocol, whereas the historical control group comprised patients treated with standard burn care alone.

Treatment allocation was not randomized but was determined by calendar period following the implementation of blue-light PBM into routine clinical practice. Patients treated between 1 January and 17 July 2024 constituted the historical control group. From 18 July 2024, blue-light PBM was incorporated into routine clinical practice as an adjunct to standard burn care. Patients treated between 18 July and 31 December 2024 constituted the PBM group. Staff training was completed before blue-light PBM was introduced into routine clinical practice on 18 July 2024. There was no transition period during which PBM was selectively offered to eligible patients. All consecutive patients who met the study eligibility criteria during each period were included.

Group allocation therefore reflected the period of care rather than individual patient or burn characteristics. Because exposure was determined by calendar time, the study was considered a retrospective historical cohort study. The same institutional burn-care protocol was applied in both periods, including the timing and criteria for clinical assessment, analgesia, wound cleansing and dressing selection, indications for enzymatic or surgical debridement, infection assessment and management, indications for autologous skin grafting, and follow-up schedules. The introduction of blue-light PBM on 18 July 2024 was the only planned change to this protocol. Treatment decisions remained individualized according to wound characteristics and clinical needs. Nevertheless, the historical comparison remains susceptible to unmeasured changes in clinical practice and other calendar-time effects.

### 2.5. Intervention

The standard wound care protocol used at the Pediatric Burn Centre included cleaning the wound, applying an advanced dressing, conservative management and postoperative care following enzymatic debridement with Nexobrid^®^ (MediWound Germany GmbH, Rüsselsheim, Germany) or surgical debridement when clinically indicated, and split-thickness skin grafting in cases where the wound was unlikely to heal spontaneously or where there was an indication for surgery. Nexobrid^®^ was selected on clinical assessment to remove necrotic tissue from deep burns. Following presentation to the Emergency Department, enzymatic debridement was performed urgently or within 24 h of the burn injury. Exact patient-level treatment times were not available in the study dataset. Advanced wound dressings available at the Burn Centre included dialkylcarbamoyl chloride (DACC) dressings, silver gelling fiber dressings, silver alginate dressings, GLG (glucose oxidase, lactoperoxidase, guaiacol) technology alginogel dressings, and silicone dressings. A polyhexamethylene biguanide (PHMB)-based cleanser was available within the institutional wound-cleansing protocol. Dressing selection was based on wound characteristics, including burn depth, exudate level, tissue condition, and stage of healing, and was reassessed throughout follow-up. After enzymatic (deep partial-thickness burns) or surgical (deep partial-thickness and full-thickness burns) debridement, following complete eschar removal, the wound bed was reassessed and managed with advanced dressing to maintain an optimal healing environment, control exudate, and promote re-epithelialization. After split-thickness skin grafting, the grafted area was protected with a non-adherent dressing according to the Burn Centre protocol, followed by scheduled dressing changes until complete graft take and wound healing. Planned dressing changes were performed every 72 h for silver gelling fiber, silver alginate, and DACC dressings, and every 24–48 h for collagenase plus sodium hyaluronate, hydrogel, and paraffin gauze. After skin grafting, the first planned dressing assessment and replacement took place approximately 5–7 days after the procedure, with subsequent changes at similar intervals according to graft and wound condition. Dressings were replaced earlier when the dressing or overlying bandage was wet or soiled. These dressing schedules were distinct from the twice-weekly PBM schedule.

The distributions of the first recorded local treatment and wound-bed preparation method by study group are reported in Supplementary Table S2. These data describe the treatments recorded for individual patients within the shared institutional care protocol. “First recorded local treatment” refers to the initial local product recorded in the study dataset; its timing relative to PHMB cleansing and PBM was not available for analysis. The date of autologous skin grafting was not available in the study dataset; therefore, the interval from admission to grafting could not be summarized or compared between groups. The products available within the institutional protocol and the first recorded local treatment were distinct descriptions of care. Recorded initial treatments also included sodium hypochlorite 0.05% antiseptic, collagenase with hyaluronate, hydrogel, and paraffin gauze, as detailed in Supplementary Table S2; availability of a product did not imply that every patient received it. These categories include antiseptics and other topical wound-care products and should not be interpreted as seven equivalent types of dressing.

Blue-light photobiomodulation was administered using the EmoLED^®^ v.1 device (Emoled srl, Florence, Italy) twice weekly before dressing application. The Class IIa, non-invasive, non-contact device uses six LED sources emitting at 400–430 nm in continuous mode. The treatment field is approximately 5 cm in diameter (approximately 20 cm^2^) at a working distance of 40 ± 10 mm. Each field was irradiated for 120 s. Wound dimensions were entered into the device; wounds exceeding a single treatment field received multiple successive applications over adjacent fields, with intentional overlap avoided. The manufacturer specifies an optical output power of 2.3 W and describes a maximum optical power density of 120 mW/cm^2^. For the study exposure duration, the latter corresponds to a nominal maximum fluence of 14.4 J/cm^2^, rather than a measured field-average fluence. Nominal emitted energy was calculated from the specified total optical power and exposure duration: 2.3 W × 120 s = 276 J per application. For N applications, nominal session energy was 276 × N J and irradiation time was 120 × N seconds. These values are nominal estimates, not measurements of delivered energy.

### 2.6. Patient Assessment

The initial clinical assessment was performed by a plastic surgeon at presentation to the pediatric Emergency Department. Burn depth was classified clinically as superficial, superficial partial-thickness, deep partial-thickness, or full-thickness according to the traditional anatomical classification [1]. The depth recorded at this initial assessment was the baseline classification used in the study database and regression models. Burn extent was estimated as a percentage of total body surface area (TBSA) using the age-adjusted Lund–Browder chart [23]. The 24 h and 72 h assessments were timed from hospital admission, rather than from the time of injury. The 24 h assessment served as the baseline for the comparative analysis of local inflammation and was completed before the first PBM exposure. Patients in the PBM group received their first treatment after the 24 h assessment. Patients in the historical control group underwent assessments at the same scheduled time points and received standard burn care alone.

The available information established the sequence of the 24 h assessment followed by the first PBM session, but did not allow precise calculation of the interval between admission and PBM initiation. The interval between injury and hospital admission was not available in the study dataset.

Subsequent PBM sessions were scheduled twice weekly. No additional routine PBM session was administered between the 24 h and 72 h assessments. The 72 h assessment therefore evaluated the early inflammatory response following the first PBM session.

The data were collected retrospectively from the referral center’s electronic medical records. The variables collected were entered into a dedicated electronic case report form (REDCap 16.0.49) and selected in accordance with the study objectives, with the aim of describing the clinical characteristics of pediatric burns, the treatments received and the associated clinical and scarring outcomes.

Local inflammatory status was assessed across four domains: erythema, exudate, pain, and pruritus. Erythema, exudate, and pruritus were graded on a four-point ordinal scale from 0 (absent) to 3 (severe), according to predefined clinical criteria. Pruritus was assessed by asking the child directly when reliable communication was possible. In younger or non-verbal children, assessment combined caregiver reports with clinical observation, including scratching and agitation. The same wound care specialist assigned the final score: 0 = absent, 1 = mild, 2 = moderate, and 3 = severe, as reported in Supplementary Table S1. This assessment was a clinical rating rather than a validated pediatric pruritus instrument.

Pain was assessed using age- and developmentally appropriate validated pediatric pain assessment tools. The Face, Legs, Activity, Cry, Consolability (FLACC) scale [24] was used in non-verbal children or in those unable to provide a reliable self-report; the Faces Pain Scale–Revised (FPS-R) [25] was used in children able to provide a reliable self-report from approximately 4 years of age; and a 0–10 Numeric Rating Scale (NRS) [26] was used in older children, generally from 8 years of age, when adequate numerical understanding was present. For inclusion in the exploratory inflammation score, the original pain scores were subsequently recoded into a common four-level ordinal variable ranging from 0 to 3. For pain, the recoded value of 0 represented the lowest category of the original instrument and did not invariably indicate complete absence of pain. Pediatric acute-pain studies have examined category thresholds separately for the FPS-R [27] and verbal NRS [28]; these findings do not validate a common recoding across instruments or its use in pediatric burns. The operational criteria and recoding procedure for all four domains are reported in Supplementary Table S1. Assessments at 24 and 72 h were performed by the same wound care specialist using the predefined scoring criteria reported in Supplementary Table S1. The assessor was not blinded to treatment exposure, as the assessments were performed as part of routine clinical care and the assessor was directly involved in patient management. The clinical assessments were recorded during routine care; pain recoding and calculation of the exploratory composite were subsequently performed for the retrospective analysis using the rules in Supplementary Table S1.

As no validated multidimensional instrument specifically combining these four domains is available for the assessment of local inflammation in pediatric burns, an exploratory composite inflammation score was calculated as the sum of the individual scores for erythema, exudate, pain, and pruritus. Each domain contributed a score ranging from 0 to 3, resulting in a total composite score ranging from 0 to 12, with higher scores indicating a greater overall burden of local inflammatory signs and symptoms. Pain scores obtained using the different age-appropriate validated instruments were recoded to the common 0–3 ordinal metric before calculation of the composite score. This harmonization allowed pain to be incorporated on the same metric as erythema, exudate, and pruritus for calculation and statistical analysis of the exploratory composite outcome, without replacing or modifying the original clinical pain assessment. The common 0–3 coding did not establish equivalence between the pain instruments, and neither the recoding nor the composite score had been validated specifically for pediatric burns. The composite score was used exclusively as an exploratory measure of the early local inflammatory response and should not be interpreted as a validated clinical scale.

Additional clinical outcomes included time to complete wound healing, defined as the number of days from hospital admission to stable epithelial coverage of all burn areas, with no residual open areas or further need for dressings to promote closure. This endpoint was determined clinically. In patients undergoing skin grafting, complete healing required healed grafted areas, closure of all remaining non-grafted burn areas, and healing of the donor sites. References to complete re-epithelialization in this study denote this endpoint. Early scar outcome was assessed using the VSS [29,30]. VSS assessment was performed approximately three months after complete wound re-epithelialization during scheduled clinical follow-up by the same plastic surgeon, who had clinical experience in assessing burn scars and associated functional limitations. The assessor was not blinded to treatment exposure because scar assessment was performed as part of routine clinical care. VSS data at the scheduled follow-up were available for all 84 patients included in the study (50/50 historical control patients and 34/34 PBM patients). The need for autologous skin grafting was also recorded. An early dressing change was defined as an unscheduled dressing replacement before the planned interval specified in Section 2.5. In this cohort, early replacement was required because dressings or overlying bandages were wet or soiled. Safety assessment was based on the retrospective review of the electronic medical records for documented treatment-related pain, adverse events, or side effects during burn treatment. Attribution to PBM was based on the clinical documentation; the safety findings therefore reflect events recorded during routine care.

For illustrative purposes, representative clinical photographs obtained during routine care were selected from one patient included in the PBM cohort to document the clinical course of wound healing following adjunctive blue-light photobiomodulation.

### 2.7. Statistical Analysis

Continuous variables were summarized as mean (SD), and categorical variables as n (%). Between-group comparisons were descriptive, using *p*-values and standardized mean differences (SMDs). The baseline composite inflammation score was compared using a two-sided Welch *t* test. Burn-agent distributions were compared using the two-sided Fisher–Freeman–Halton exact test because some categories contained few patients.

Anatomical sites were summarized as separate binary variables because individual burns could involve multiple sites. Between-group comparisons for each recorded site used two-sided Fisher’s exact tests and standardized mean differences. These comparisons were descriptive, and no adjustment for multiple testing was applied.

The composite inflammation score was analyzed as a continuous outcome. The 24 h composite score was used as the baseline inflammatory assessment. Domain-specific change scores were calculated analogously for erythema, exudate, pain and pruritus. The primary analysis used an analysis-of-covariance linear regression model with the 72 h composite inflammation score as the dependent variable and PBM as the main exposure. The model adjusted for the 24 h composite inflammation score, age, sex, total body surface area (TBSA), burn depth, burn agent and Nexobrid^®^ treatment.

A sensitivity analysis additionally adjusted for the first recorded local treatment, entered as a categorical variable with the seven categories reported in Supplementary Table S2. This analysis assessed whether the association between PBM and the 72 h inflammation score persisted after accounting for differences in initial local treatment. The timing of this treatment relative to PBM was not available in the study dataset; therefore, this analysis was interpreted as an exploratory adjustment for recorded care rather than as an estimate of a causal treatment effect.

Regression coefficients were reported with 95% confidence intervals and *p*-values. Heteroskedasticity-consistent HC3 robust standard errors were used for linear-regression inference. For these inflammation models, confidence intervals and *p*-values were calculated using the t distribution with the residual degrees of freedom. The singleton thermal electrical-arc category fitted its observation exactly. HC3 inference for the PBM–historical control contrast was therefore calculated from the equivalent regression on the remaining 83 patients, with the singleton category indicator omitted.

The 24 h-to-72 h change in composite inflammation score was summarized descriptively to support clinical interpretation.

Secondary continuous outcomes, including healing time and total VSS score, were analyzed using linear regression models.

The healing-time model included PBM exposure, age, sex at birth, TBSA, burn depth, burn agent, and Nexobrid^®^ treatment. Age and TBSA were entered as continuous linear terms, and categorical variables were represented by indicator variables. The historical control group was the reference exposure. The outcome was the number of days from hospital admission to complete re-epithelialization. Results were reported as adjusted mean differences in days, with HC3 robust standard errors, 95% confidence intervals, and two-sided *t* tests using the residual degrees of freedom. All 84 patients had complete data. The singleton thermal electrical-arc category fitted its observation exactly; HC3 inference for the PBM–historical control contrast was therefore calculated from the equivalent regression on the remaining 83 patients, omitting the singleton category indicator.

The main VSS model included PBM exposure, age, sex at birth, TBSA, burn depth, burn agent, and Nexobrid^®^ treatment. Age and TBSA were entered as continuous linear terms, and categorical variables were represented by indicator variables. The historical control group was the reference exposure. A sensitivity model additionally included the nine anatomical-site indicators reported in Table 1, allowing each patient to have multiple affected sites. A further sensitivity model also included autologous skin grafting. Grafting was included only in sensitivity analysis because it was a treatment-course variable potentially influenced by PBM and could lie on the pathway between treatment and scar outcome. These analyses were not intended to estimate causal direct or mediated effects. Results were reported as adjusted mean differences in VSS points, with HC3 robust standard errors, 95% confidence intervals, and two-sided *t* tests using the residual degrees of freedom.

All 84 patients had complete data. The single thermal electrical-arc burn constituted a separate burn-agent category and was fitted exactly by its category indicator. Consequently, this observation provided no information for the PBM–historical control contrast. HC3 inference for this contrast was calculated from the equivalent regression on the remaining 83 patients, omitting the singleton category indicator.

Early dressing change and autologous skin grafting were reanalyzed as exploratory binary outcomes using logistic regression. Crude odds ratios (ORs) were estimated using maximum-likelihood logistic regression, with Wald 95% confidence intervals (CIs) and two-sided Wald tests. Adjusted models included age, sex at birth, total body surface area (TBSA), burn depth, burn agent, and Nexobrid^®^ treatment, with the historical control group as the reference exposure. Age and TBSA were entered as continuous linear terms, and all five recorded burn-agent categories were retained. Nexobrid^®^ treatment was coded against dermabrasion. Because the single electrical-burn observation caused quasi-complete separation in the conventional adjusted models, adjusted ORs were estimated using Firth’s bias-reduced logistic regression, which maximizes the binomial log-likelihood with a penalty equal to one-half the log determinant of the expected Fisher information matrix, with profile penalized-likelihood 95% CIs and penalized likelihood-ratio tests. Both models included all 84 complete cases, without imputation or data-driven covariate selection. Tests were two-sided, with no adjustment for multiple comparisons. These binary-outcome reanalyses were performed using Python 3.12.14, statsmodels 0.14.6 for conventional logistic regression, and a custom implementation of Firth’s penalized likelihood using SciPy 1.18.1 and NumPy 2.5.3.

Individual inflammatory domains were reanalyzed as exploratory secondary endpoints using separate linear regression models for erythema, exudate, pain, and pruritus. Each model used the 72 h domain score as the dependent variable and included PBM exposure, the corresponding 24 h domain score, age, sex, TBSA, burn depth, burn agent, and Nexobrid^®^ treatment. Results were expressed as adjusted mean differences between PBM and historical control in points on the 0–3 scale. HC3 robust standard errors and two-sided *t* tests with residual degrees of freedom were used. The singleton thermal electrical-arc category was handled as described for the composite inflammation models. No adjustment for multiple testing was applied.

Of the 137 patients assessed for eligibility, 53 were excluded during cohort selection for the reasons reported in Section 2.2 and Figure 1. Among the 84 included patients, no data were missing for the outcomes or covariates used in the reconstructed analyses, and no imputation was performed. Descriptive analyses included all 84 patients: 50 historical controls and 34 PBM patients. Both unadjusted logistic models and both adjusted Firth logistic models included all 84 patients, with 34 early dressing-change events and 32 grafting events.

For the composite inflammation model and its initial-treatment sensitivity analysis, the four individual inflammatory-domain models, the healing-time model, and the three VSS models, all 84 patients had complete data. However, the singleton thermal electrical-arc category fitted its observation exactly and contributed no information to the adjusted PBM–historical control contrast. HC3 confidence intervals and *p*-values for these linear-model contrasts were therefore calculated using the equivalent regressions on 83 patients: 49 historical control and 34 PBM patients. This was a consequence of the model specification, not an exclusion due to missing data.

The statistical workflow used R version 4.5.2 (R Foundation for Statistical Computing, Vienna, Austria) and Python. The software and packages used for the binary-outcome reanalyses are specified above. The baseline Welch *t* test was calculated using SciPy 1.18.1.

## 3. Results

### 3.1. Patients

A total of 84 pediatric patients were enrolled in the study. The mean age of the patients was about 8 years (0–17) with a prevalence of female sex (55 subjects—65.5%). Sixty-one patients (72.6%) had deep partial-thickness burns and 23 (27.4%) had full-thickness burns. The most frequent burn agent was scalding liquid (64.3%), followed by flame (15.5%) and contact (14.3%).

The mean burn area at admission was 14.3% TBSA. Thirty-four patients underwent PBM treatment with blue light, while 50 patients were in the historical control group. Forty-four patients required advanced enzymatic debridement (52.4%), and 32 underwent autologous grafting (38.1%). Ten patients received both Nexobrid^®^ and autologous grafting (8 historical controls and 2 PBM patients); 34 received Nexobrid^®^ without grafting, 22 underwent dermabrasion followed by grafting, and 18 underwent dermabrasion without grafting. These mutually exclusive treatment combinations are reported in Supplementary Table S2. Descriptive group comparisons are reported in Table 1.

### 3.2. Inflammatory Response

The exploratory composite local inflammation score summarized four clinical domains—erythema, exudate, pain, and pruritus—each scored from 0 to 3, for a total ranging from 0 to 12. At the pre-exposure 24 h assessment, the mean score was 8.9 ± 1.6 in the historical control group and 7.6 ± 1.5 in the PBM group (*p* < 0.001; Table 1). This baseline imbalance was considered when interpreting subsequent between-group differences. The primary model compared 72 h scores with adjustment for the corresponding 24 h score and other clinical covariates; baseline adjustment was not conditional on the statistical significance of the initial comparison.

The mean 72 h composite inflammation score was 6.2 ± 2.0 in the historical control group and 3.3 ± 1.7 in the PBM group (Figure 2). After adjustment for the 24 h composite inflammation score, age, sex, TBSA, burn depth, burn agent, and Nexobrid^®^ treatment, PBM was associated with a lower 72 h score (adjusted mean difference −2.70 points, 95% CI −3.71 to −1.68; *p* < 0.001). In the sensitivity analysis, additionally adjusting for the first recorded local treatment, the estimated difference was attenuated but remained statistically significant (adjusted mean difference −1.88 points, 95% CI −2.96 to −0.80; *p* < 0.001).

The change in the inflammation score between 24 and 72 h was greater in the PBM group than in the historical control group (4.4 ± 2.1 vs. 2.7 ± 2.3). This descriptive reduction is shown in Figure 3 to support clinical interpretation; formal inference for early inflammation is based on the baseline-adjusted 72 h model reported above. The composite inflammation score increased between 24 and 72 h in four patients, all in the historical control group.

### 3.3. Wound Healing Time

The healing time for pediatric burns was analyzed on the basis of the number of days elapsed between the patient’s admission and the complete wound healing as defined in Section 2.6, including donor-site healing in grafted patients. The average time for complete healing for the PBM group was approximately 7.5 days shorter than for the historical control group (29.4 ± 5.7 vs. 36.9 ± 7.4). After adjustment for age, sex, TBSA, burn depth, burn agent, and Nexobrid^®^ treatment, PBM was associated with a shorter time to complete re-epithelialization (adjusted mean difference −5.98 days, 95% CI −8.68 to −3.28; *p* < 0.001). Individual healing times and group means with standard deviations are shown in Figure 4.

Figure 5 and Figure 6 show the clinical course of two representative patients included in the PBM group. The photographic sequence illustrates wound-bed preparation, PBM application, dressing placement, and complete re-epithelialization at 21 days after the first dermabrasion, with restoration of skin integrity.

### 3.4. Individual Inflammatory Domains

Observed domain scores and adjusted between-group differences are reported in Table 2. After adjustment for the corresponding 24 h domain score, age, sex, TBSA, burn depth, burn agent, and Nexobrid^®^ treatment, PBM was associated with lower 72 h scores for erythema, exudate, pain, and pruritus. Adjusted mean differences ranged from −0.59 points for pruritus to −0.85 points for erythema on the 0–3 scale. These findings were exploratory, and *p*-values were not adjusted for multiple testing.

### 3.5. Scar Outcome

Early scar outcome was assessed using the Vancouver Scar Scale (VSS) approximately three months after complete wound re-epithelialization. The VSS evaluates four scar characteristics—vascularity (0–3), pigmentation (0–2), pliability (0–5), and height (0–3)—yielding a total score ranging from 0 to 13, with higher scores indicating greater scar severity. VSS data were available for all 84 patients included in the study (50/50 historical control patients and 34/34 PBM patients); therefore, no patients were excluded from the scar outcome analysis because of missing VSS data.

At this follow-up, the mean total VSS score was 7.4 ± 2.1 in the historical control group and 5.6 ± 1.9 in the PBM group (Figure 7). After adjustment for age, sex, TBSA, burn depth, burn agent, and Nexobrid^®^ treatment, PBM was associated with a lower total VSS score (adjusted mean difference −1.46 points, 95% CI −2.46 to −0.47; *p* = 0.005). The association remained after additional adjustment for anatomical sites (adjusted mean difference −1.36 points, 95% CI −2.63 to −0.10; *p* = 0.035) and after further adjustment for autologous skin grafting (adjusted mean difference −1.36 points, 95% CI −2.63 to −0.08; *p* = 0.037). These sensitivity analyses were exploratory and should be interpreted cautiously given the number of covariates relative to the sample size.

### 3.6. Early Dressing Changes and Autologous Skin Graft Requirement

Early dressing change was required in 26/50 patients in the historical control group (52.0%) and 8/34 patients in the PBM group (23.5%). The crude OR for PBM versus historical control was 0.28 (95% CI 0.11–0.75; *p* = 0.011). After adjustment for age, sex, TBSA, burn depth, burn agent, and Nexobrid^®^ treatment using Firth’s logistic regression, PBM was associated with lower odds of early dressing change (adjusted OR 0.25, 95% CI 0.07–0.79; *p* = 0.017). In the historical control group, the 52.0% frequency reflected the need to replace wet or soiled dressings or bandages before the planned interval; it should not be interpreted as an infection rate.

Autologous skin grafting was performed in 20/50 patients in the historical control group (40.0%) and 12/34 patients in the PBM group (35.3%). The crude OR was 0.82 (95% CI 0.33–2.02; *p* = 0.663). The adjusted analysis did not show a statistically significant association between PBM and grafting requirement (adjusted OR 0.63, 95% CI 0.17–2.16; *p* = 0.462). Both adjusted models included all 84 patients, with 34 early dressing-change events and 32 grafting events.

### 3.7. Safety

No treatment-related pain, adverse events, or side effects attributable to blue-light photobiomodulation were documented in the clinical records reviewed during the study period. This finding describes recorded tolerability and does not exclude events that were not documented during routine care.

## 4. Discussion

Pediatric burn care remains challenging because treatment aims not only to achieve rapid wound closure but also to reduce pain, prevent infection, preserve function, and minimize long-term scarring. Achieving these goals requires timely wound assessment, appropriate local management, advanced dressings, surgery when necessary, and close multidisciplinary collaboration [31,32,33,34]. Among the many factors that influence burn healing, the early inflammatory response plays a central role, as persistent inflammation can delay re-epithelialization and increase the risk of abnormal scar formation.

Compared with historical controls, children receiving adjunctive PBM had lower baseline-adjusted inflammation scores at 72 h, shorter healing times, and lower VSS scores approximately three months after complete re-epithelialization. PBM was also associated with fewer unplanned dressing changes. Early replacement in this cohort was prompted by wet or soiled dressings or bandages; the 52.0% frequency among historical controls therefore describes this care requirement, rather than infection incidence. Although autologous skin grafting was numerically less frequent in the PBM group, the adjusted analysis did not show a statistically significant association with grafting requirement (adjusted OR 0.63, 95% CI 0.17–2.16; *p* = 0.462). These associations were observed across several outcomes, but the study cannot establish whether they reflect a treatment effect, residual confounding, or both.

Inflammation is indispensable for initiating tissue repair after a burn injury, yet its persistence rapidly becomes detrimental. Once the initial protective response has fulfilled its role, failure to restore inflammatory homeostasis promotes continuous recruitment of inflammatory cells, sustained cytokine production and excessive oxidative stress. The consequence is a wound environment that delays progression toward the proliferative phase, impairs keratinocyte migration and favors the development of fibrotic rather than regenerative repair. For this reason, successful burn healing depends not only on mounting an adequate inflammatory response but also on resolving it at the appropriate time [35].

Lower scores were observed across all four clinical domains. However, the exploratory composite combined clinical signs and symptoms and did not directly measure inflammatory pathways or biomarkers. The findings therefore describe differences in the recorded clinical response rather than demonstrate a specific biological mechanism.

Experimental studies have shown that blue light can generate reactive oxygen species in cellular systems [10], while studies of superficial animal wounds have reported changes in inflammatory infiltrates, re-epithelialization, and collagen organization after blue-light exposure [11,12,13]. These effects depend on exposure parameters, the experimental model, and the time of assessment; they should not be interpreted as uniform suppression of inflammation. In a murine model, blue-light PBM was associated with an early increase in inflammatory-cell responses followed by changes in angiogenesis and tissue organization [13]. Such findings provide a biological rationale for studying early inflammatory modulation, but they cannot establish the mechanism underlying the clinical associations in this pediatric cohort. The present study measured clinical signs and symptoms rather than cellular or molecular responses; it therefore cannot determine which biological mechanisms contributed to the observed associations.

The individual components of the exploratory score help describe the clinical pattern observed in this cohort. Persistent erythema and excessive wound exudate are commonly interpreted as signs of ongoing inflammatory activity and delayed stabilization of the wound bed. Excessive exudation may also compromise dressing performance, promote peri-wound maceration and increase the need for additional wound manipulation, whereas prolonged erythema reflects sustained vascular activation within injured tissues [36,37]. The concurrent reduction in both parameters in the PBM group is consistent with a wound environment more favorable to tissue repair, without demonstrating a treatment mechanism.

Pain followed a similar trend. In pediatric burn care, pain is influenced not only by tissue damage itself but also by inflammatory mediator release, repeated dressing procedures and the emotional distress associated with treatment [38]. Lower pain scores were observed in the PBM group, but analgesic consumption was not systematically documented. The study therefore cannot determine the contribution of concomitant analgesia or establish an inflammatory or nociceptive mechanism for this association.

Pruritus should also be interpreted within the same biological context. While often regarded as a late consequence of burn healing, pruritus frequently emerges during active re-epithelialization and reflects persistent interactions between inflammatory and neural pathways. In children, uncontrolled pruritus may interfere with wound care, encourage scratching and contribute to repeated mechanical injury of newly regenerated tissue [39,40]. Lower recorded pruritus scores were observed in the PBM group, but this association does not establish an effect on inflammatory or neural pathways.

A further finding was the shorter time to complete re-epithelialization observed in the PBM group. Complete re-epithelialization occurred approximately one week earlier than in patients receiving standard care alone, and this association remained significant after adjustment for the principal clinical confounders. Such a difference is unlikely to be trivial in pediatric burn management. Time to wound closure is one of the strongest predictors of subsequent clinical outcome, influencing the duration of treatment, susceptibility to infection and, ultimately, the risk of hypertrophic scar formation [2]. Earlier epithelial restoration may influence the conditions under which tissue remodeling proceeds, although this biological relationship was not directly tested in the present study [4].

Lower early inflammation scores, shorter healing times, and lower VSS scores were observed in the PBM group. The study did not establish whether these associations were causally related or whether early changes in inflammation mediated subsequent outcomes.

The current understanding of burn scar pathophysiology states that the inflammatory response not only initiates tissue repair but also shapes the activity of fibroblasts, the composition of the extracellular matrix and the organization of newly deposited collagen. When inflammatory signaling persists beyond necessity, fibroblast activation becomes prolonged, collagen turnover is altered and the wound is more likely to evolve toward excessive fibrosis. Conversely, timely resolution of inflammation creates conditions that favor a more physiological remodeling process [3,4,5,41].

Lower VSS scores were observed approximately three months after complete re-epithelialization. Scar formation and remodeling involve overlapping biological processes that continue after wound closure [42,43,44,45]. This finding concerns early scar characteristics; the study did not assess mature scars or subsequent scar evolution. The observed associations do not establish that early inflammatory changes caused the differences in healing time or VSS scores.

At the same time, these findings should be interpreted with appropriate caution. Scar formation is a multifactorial process influenced by burn depth, anatomical location, genetic predisposition, mechanical forces and surgical management [41]. The association with early VSS scores persisted in the sensitivity analyses, although residual confounding and the observational design preclude causal attribution to PBM. The association observed should therefore be considered hypothesis-generating and warrants confirmation in prospective randomized studies incorporating longer follow-up and serial scar evaluations.

No adverse events attributable to photobiomodulation were documented during the study period. This observation supports the tolerability recorded in this cohort, but the small sample and retrospective reliance on routine clinical records limit conclusions about safety. The device is non-invasive and non-contact and was used as an adjunct during the dressing pathway, consistent with previous pediatric experience [21]. The study did not establish superior safety, quantify procedural discomfort or treatment complexity, or exclude uncommon or undocumented adverse events. Prospective studies should assess these outcomes systematically.

The study was limited by its small sample size and unequal group sizes (34 PBM patients and 50 historical control patients), which limited the precision of the estimates, and by the short scar follow-up of approximately three months after complete re-epithelialization, which did not allow assessment of long-term scar outcomes. Selection bias may also have arisen from requiring sufficiently complete clinical records and outcome data for inclusion. Patients excluded because of incomplete documentation or missing outcomes may have differed systematically from those included in terms of burn severity, treatment course, or healing outcomes. The dataset did not contain instrument-specific pain counts, confirmation of instrument continuity between 24 and 72 h, or assessment timing relative to analgesia and dressing procedures; these factors could not be evaluated. Selection of complete cases may have affected the observed associations and limits the generalizability of the findings to the broader pediatric burn population. The dataset contained total burn TBSA and a categorical depth classification, but not separate TBSA estimates for each depth component. Differences in the extent of deep partial-thickness and full-thickness injury within individual burns could therefore not be assessed.

Treatment exposure was determined by calendar period, so the association with PBM could not be separated from other differences between the two periods of care. Use of a shared institutional protocol does not exclude changes in staff experience, documentation, treatment selection, or other aspects of practice. Adjustment for recorded covariates cannot eliminate these sources of confounding. Inflammatory assessments were performed by the same wound care specialist, and VSS assessments by the same plastic surgeon. Neither assessor was blinded to treatment exposure. Using consistent assessors did not eliminate the risk of measurement bias.

Although both cohorts were managed under the same institutional protocol, the distribution of initial local treatments differed between groups and may have contributed to the observed outcome differences. Sodium hypochlorite was recorded in 24.0% of historical control patients and 2.9% of PBM patients, hydrogel in 12.0% and 32.4%, and DACC in 24.0% and 8.8%, respectively (Supplementary Table S2). Additional adjustment for the first recorded local treatment attenuated the adjusted difference in the 72 h inflammation score from −2.70 to −1.88 points, although the association remained statistically significant. This sensitivity analysis concerned the 72 h inflammation outcome and does not establish adequate control of concomitant local treatments in the healing-time or VSS analyses. Residual confounding related to local treatment selection cannot be excluded. Grafting timing was not available in the study dataset, preventing assessment of differences in the timing of this procedure between groups.

Prospective studies should determine whether the observed associations persist under randomized allocation and blinded outcome assessment. Serial biological measurements would be needed to investigate inflammatory mechanisms, while longer follow-up would be required to assess mature scar outcomes.

## 5. Conclusions

In this retrospective historical cohort of pediatric patients with deep thermal burns, adjunctive blue light photobiomodulation was associated with a more favorable early inflammatory response, shorter time to complete re-epithelialization, and better early scar quality compared with standard burn care alone. Lower scores were observed at 72 h across all four clinical domains, although the composite was exploratory and the study did not establish a mechanism linking these differences to subsequent healing. Patients receiving PBM also required fewer unplanned dressing changes, whereas no significant difference was observed in the requirement for autologous skin grafting.

The scar findings were limited to an early assessment approximately three months after complete re-epithelialization and do not establish a long-term benefit. No adverse events attributable to PBM were documented in the available clinical records.

Given the retrospective design, historical group allocation, exploratory inflammatory score, and relatively short scar follow-up, these findings should not be interpreted as evidence of a causal treatment effect. Prospective multicenter randomized studies with standardized inflammatory assessment, longer scar follow-up, and predefined clinical endpoints are required to confirm these observations and to establish the role of blue light photobiomodulation in pediatric burn care.

## Figures and Tables

**Figure 1:**
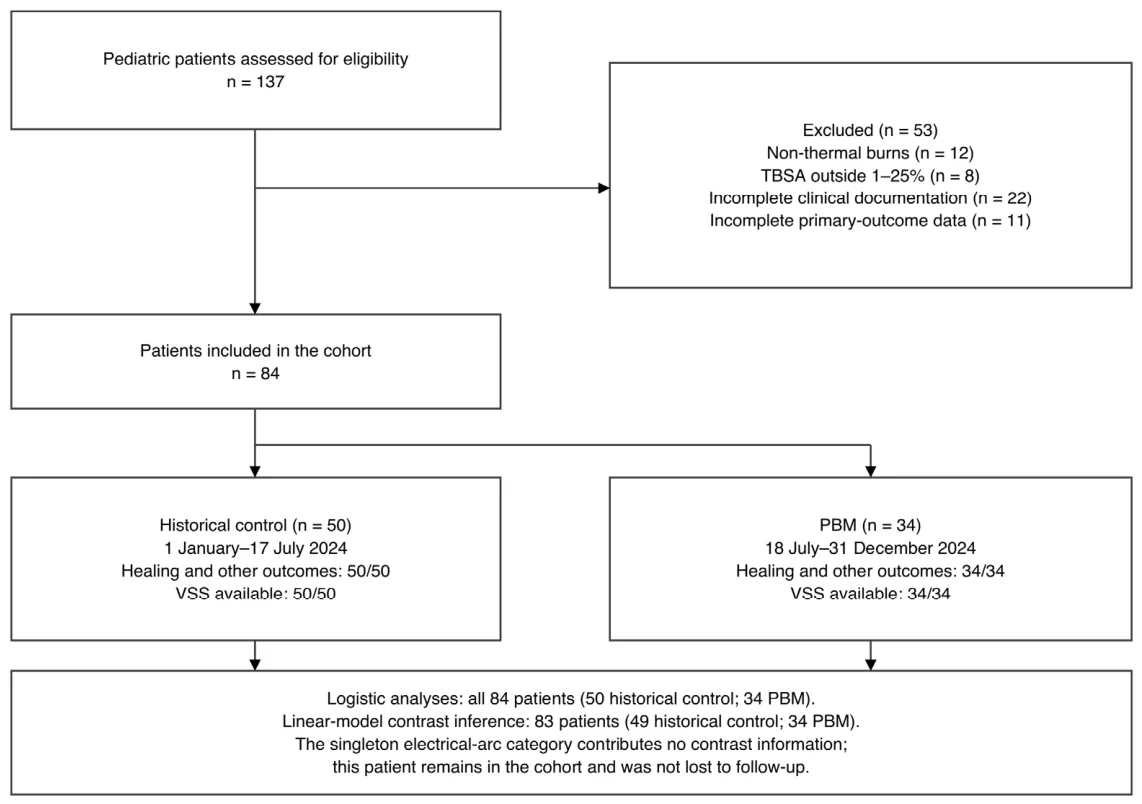
Flow diagram of participant selection and cohort allocation.

**Figure 2:**
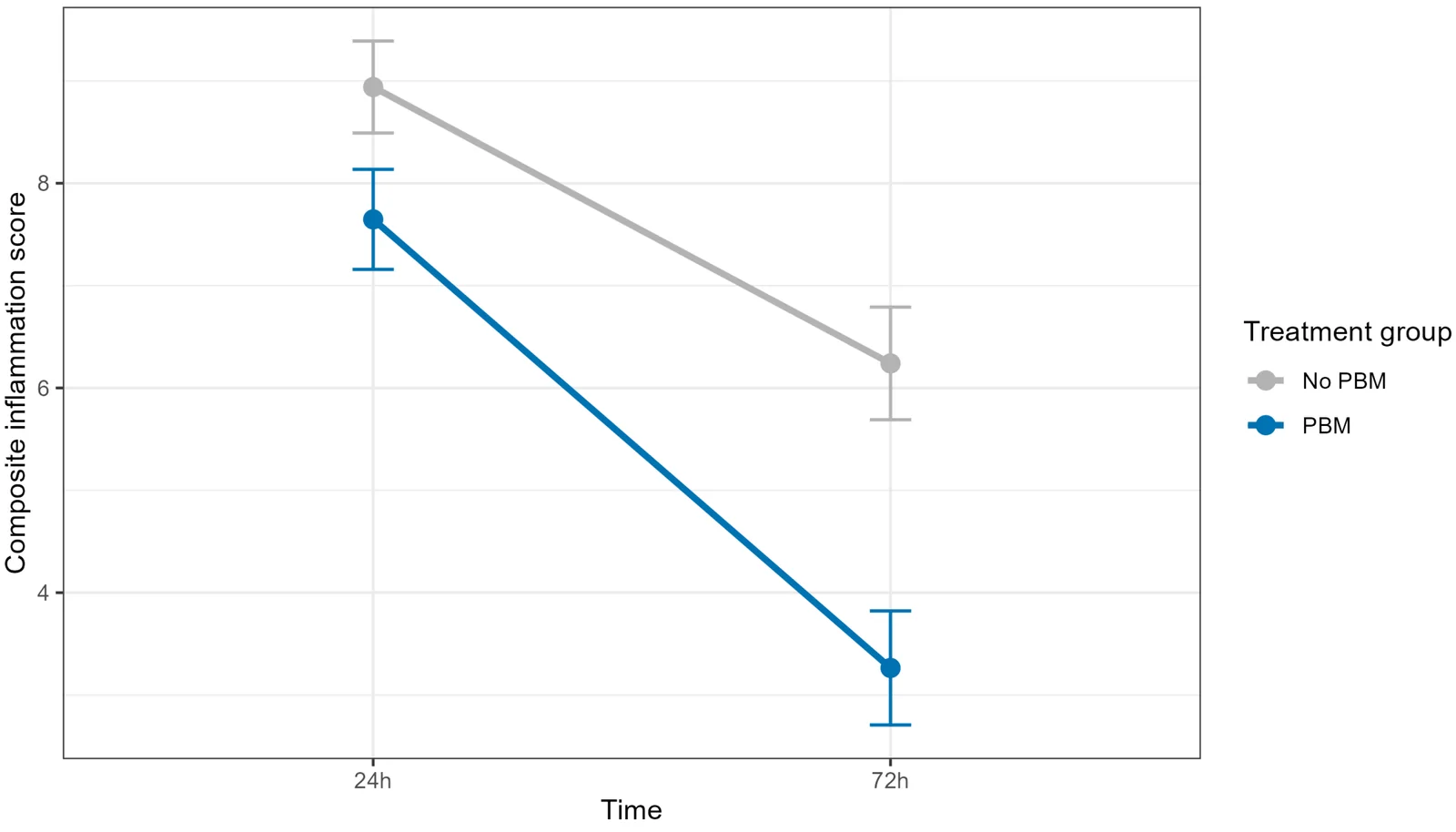
Composite inflammation score at baseline/24 h and 72 h by treatment group. Group means are shown with approximate 95% confidence intervals. “No PBM” denotes the historical control group.

**Figure 3:**
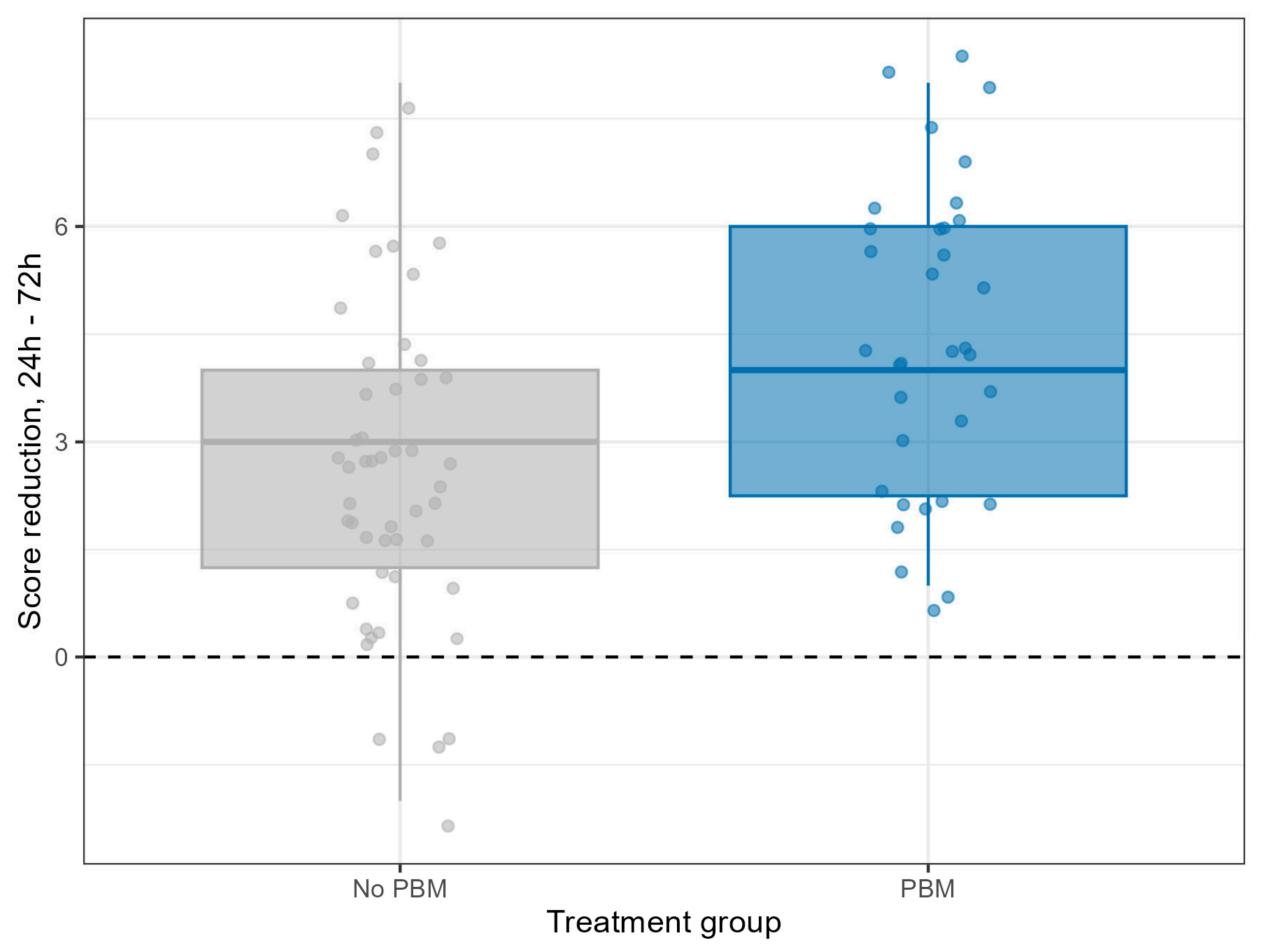
Composite inflammation score reduction from baseline/24 h to 72 h. The bold horizontal lines indicate the medians of the observed data. Positive values indicate reduced inflammation. “No PBM” denotes the historical control group.

**Figure 4:**
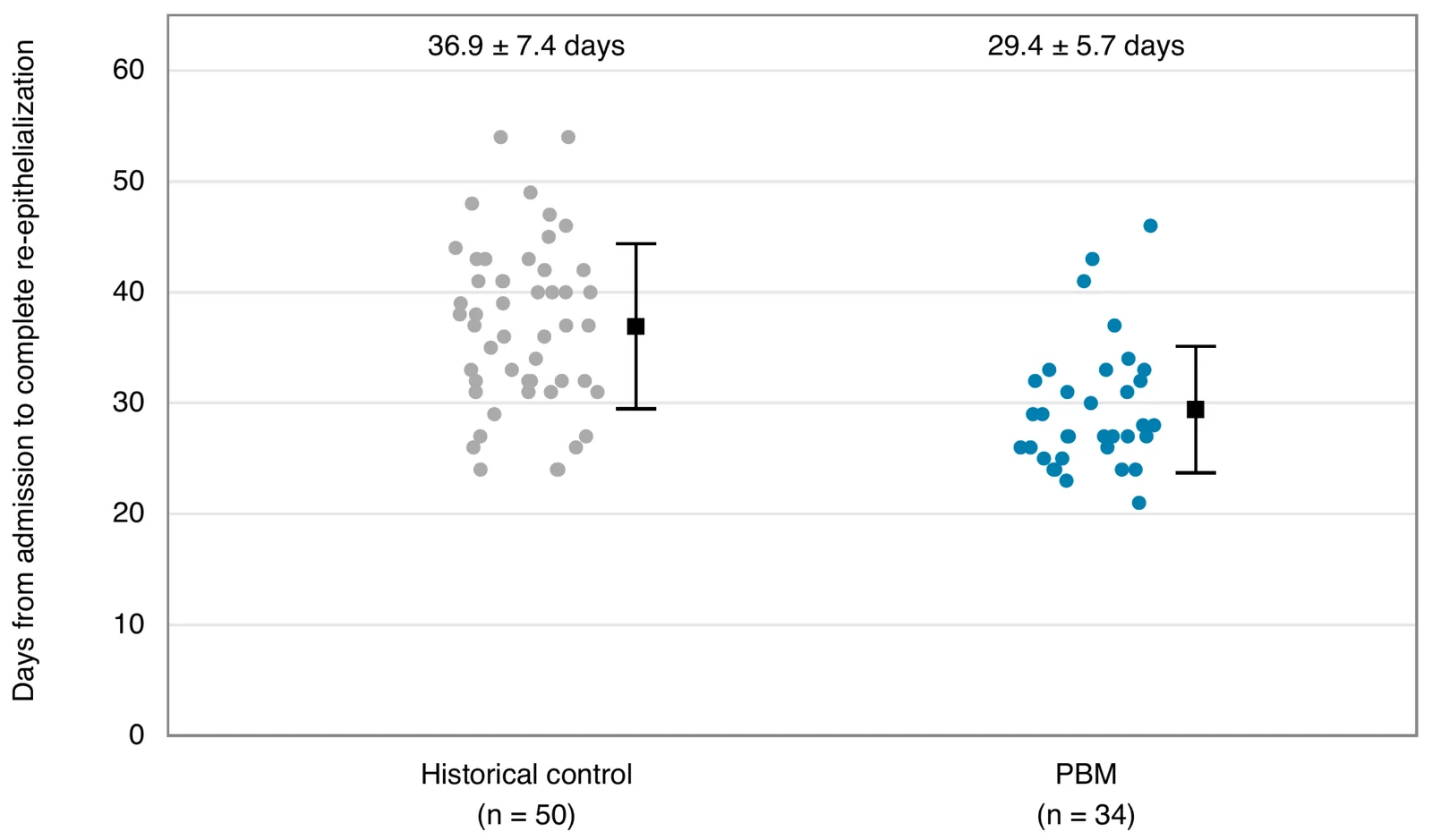
Observed time from hospital admission to complete re-epithelialization, including donor-site healing where applicable, in the historical control (n = 50) and PBM (n = 34) groups. Each point represents one patient; horizontal displacement is used only to distinguish overlapping observations. Black squares and bars show the mean ± SD: 36.9 ± 7.4 and 29.4 ± 5.7 days, respectively. The adjusted mean difference was −5.98 days (95% CI −8.68 to −3.28; *p* < 0.001), from the regression model described in Section 2.7.

**Figure 5:**
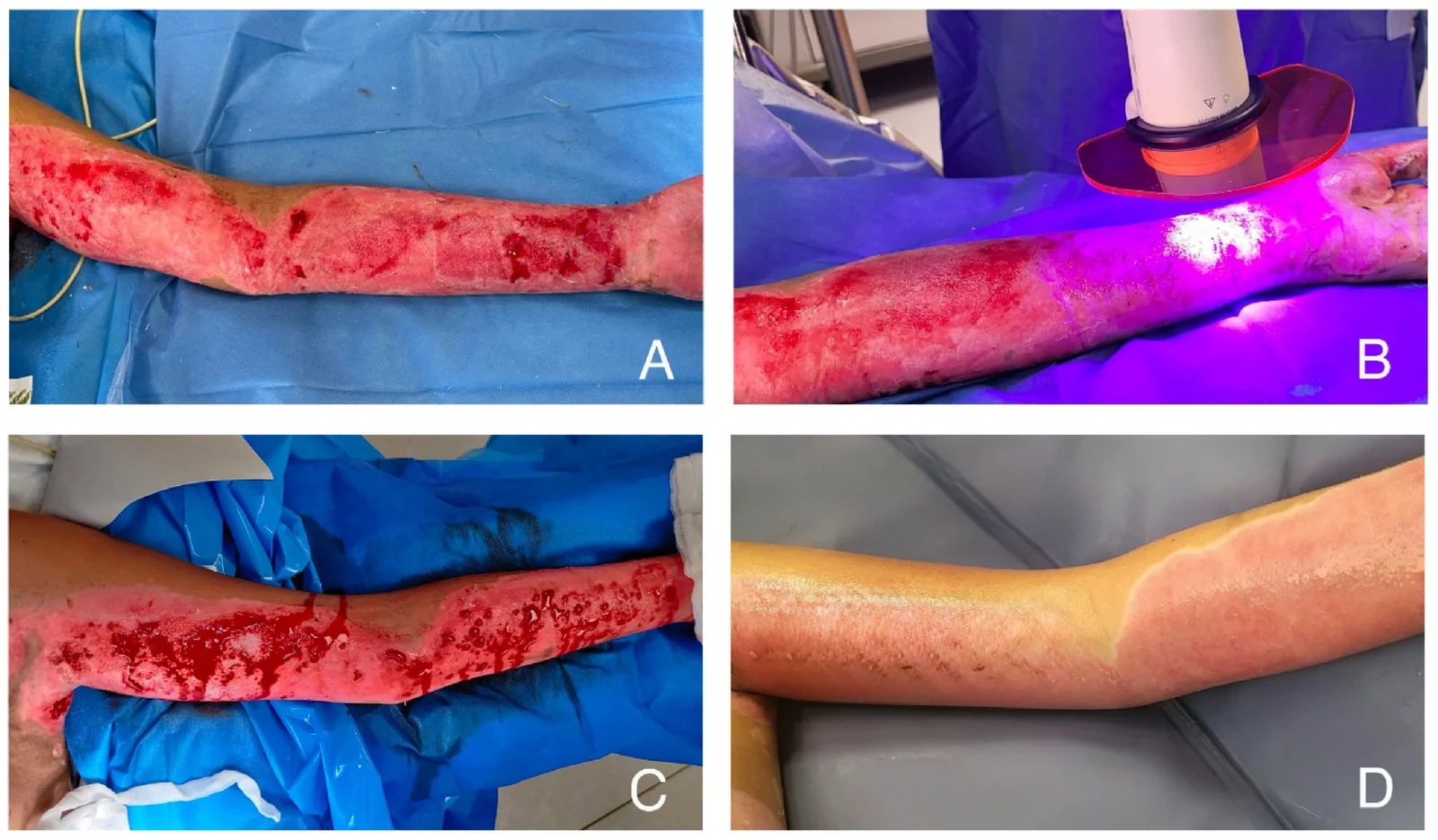
Representative case of a 12-year-old girl with burns involving >20% of the total body surface area (TBSA), including an extensive deep partial-thickness burn of the upper limb, treated with blue light photobiomodulation (PBM). The deep partial-thickness classification was based on the plastic surgeon’s clinical assessment at initial presentation to the pediatric Emergency Department. The first dermabrasion, performed within 24 h of the burn injury, defines day 0 for this photographic sequence. (**A**) Wound appearance following initial dermabrasion. (**B**) Application of blue-light PBM (EmoLED^®^) after the baseline assessment and before dressing placement. (**C**) Wound coverage with a dialkylcarbamoyl chloride (DACC) dressing and a silicone layer at 72 h after the first dermabrasion. (**D**) Clinical appearance with complete re-epithelialization at 21 days after the first dermabrasion. Informed consent was obtained for publication of the clinical photographs.

**Figure 6:**
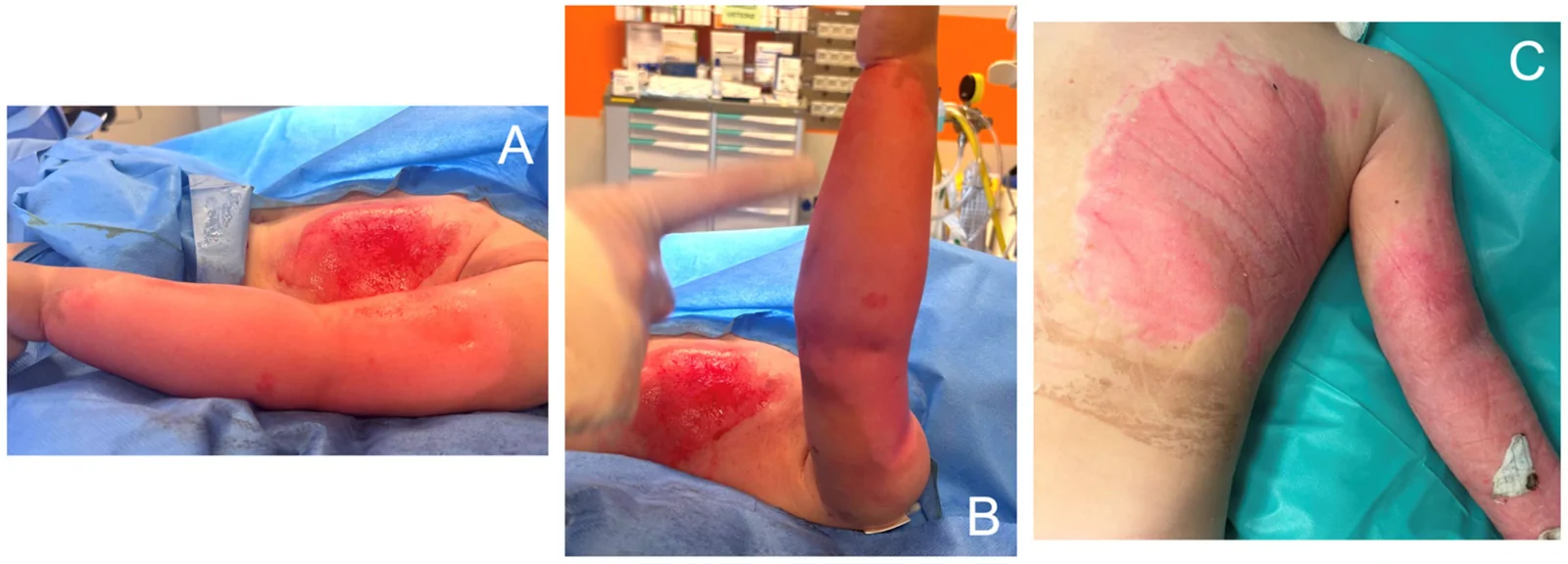
Representative case of a deep second-degree (deep partial-thickness) scald burn of the trunk and upper limb in a 6-year-old boy, involving 16% of total body surface area (TBSA). Burn depth was assessed clinically by a plastic surgeon on presentation to the Emergency Department. Dermabrasion was performed within 24 h of injury, followed by the first application of blue-light photobiomodulation (PBM). (**A**,**B**) Burn areas after dermabrasion on day 0. The patient received four PBM applications of 120 s each at 4-day intervals, with alginogel and paraffin gauze dressings. (**C**) Complete re-epithelialization at day 21.

**Figure 7:**
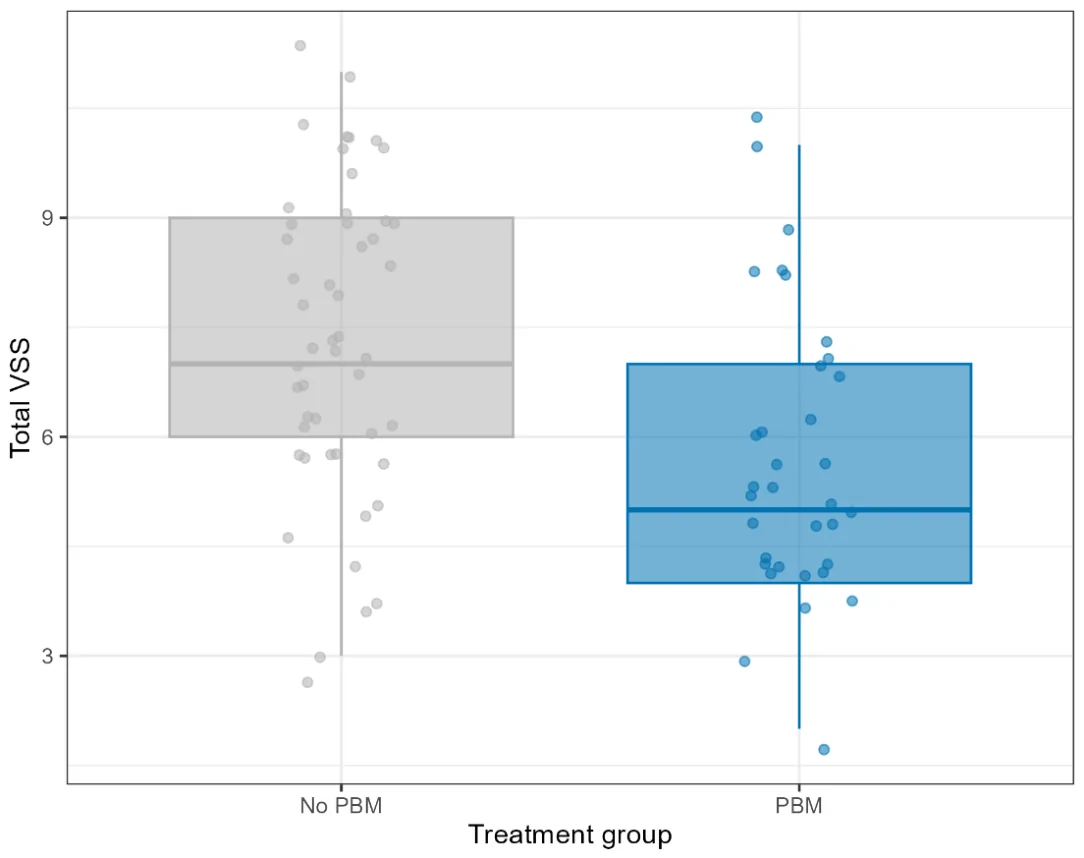
Comparison of total VSS score between the group of patients who received standard therapy and the group who also received PBM treatment. The bold horizontal lines indicate the medians of the observed data. “No PBM” denotes the historical control group.

**Table 1:** Descriptive comparison between historical control and PBM groups.

Variable	Overall	Historical Control	PBM Group	*p*-Value	SMD
n	84	50	34		
Age, years, mean (SD)	8.4 (4.5)	8.2 (4.6)	8.8 (4.3)	0.521	0.144
Weight, kg, mean (SD)	34.2 (13.9)	34.2 (14.1)	34.2 (13.8)	0.990	0.003
Sex, n (%)				0.410	0.235
Male	29 (34.5)	15 (30.0)	14 (41.2)		
Female	55 (65.5)	35 (70.0)	20 (58.8)		
Burn agent, n (%)				0.120	0.626
Contact	12 (14.3)	8 (16.0)	4 (11.8)		
Flame	13 (15.5)	11 (22.0)	2 (5.9)		
Scalding liquid	54 (64.3)	27 (54.0)	27 (79.4)		
Boiled oil	4 (4.8)	3 (6.0)	1 (2.9)		
Thermal electrical-arc burn	1 (1.2)	1 (2.0)	0 (0.0)		
Burn depth, n (%)				1.000	0.034
Deep partial-thickness	61 (72.6)	36 (72.0)	25 (73.5)		
Full-thickness	23 (27.4)	14 (28.0)	9 (26.5)		
TBSA (%), mean (SD)	14.3 (5.7)	14.3 (5.6)	14.4 (5.8)	0.979	0.006
Anatomical site, n (%)					
Face/neck	2 (2.4)	2 (4.0)	0 (0.0)	0.512	0.289
Chest	51 (60.7)	32 (64.0)	19 (55.9)	0.500	0.166
Abdomen	43 (51.2)	25 (50.0)	18 (52.9)	0.827	0.059
Back	20 (23.8)	9 (18.0)	11 (32.4)	0.191	0.335
Upper limb(s)	20 (23.8)	13 (26.0)	7 (20.6)	0.612	0.128
Hands	2 (2.4)	2 (4.0)	0 (0.0)	0.512	0.289
Pubic region	12 (14.3)	7 (14.0)	5 (14.7)	1.000	0.020
Thighs	37 (44.0)	18 (36.0)	19 (55.9)	0.079	0.407
Feet	8 (9.5)	3 (6.0)	5 (14.7)	0.260	0.289
TBSA, n (%)				0.954	0.069
<10%	11 (13.1)	7 (14.0)	4 (11.8)		
10–19%	63 (75.0)	37 (74.0)	26 (76.5)		
>=20%	10 (11.9)	6 (12.0)	4 (11.8)		
Nexobrid^®^ wound-bed preparation, n (%)	44 (52.4)	26 (52.0)	18 (52.9)	1.000	0.019
Pre-exposure composite inflammation score at 24 h, mean (SD)	—	8.9 (1.6)	7.6 (1.5)	<0.001	—

(SMD: Standardized Mean Difference; SD: Standard Deviation). Note: The *p*-value for burn agent was calculated using the two-sided Fisher–Freeman–Halton exact test. Anatomical sites were recorded as non-mutually exclusive categories. Each patient was counted once in every affected site; therefore, percentages may sum to more than 100%. Percentages were calculated using the total number of patients in each group. Site-specific *p*-values were calculated using two-sided Fisher’s exact tests, and SMDs were calculated for the presence versus absence of each recorded site. For the pre-exposure composite inflammation score, the *p*-value was calculated using a two-sided Welch *t* test. Group summaries and the *p*-value are reported; the overall summary and SMD are not reported (—).

**Table 2:** Observed inflammatory domain scores, descriptive reductions, and adjusted PBM–historical control differences at 72 h.

Domain	Group	24 h Mean	72 h Mean	Reduction, Points (%)	Adjusted Difference, Points	95% CI	*p*-Value
**Erythema**	Historical control	2.40	1.60	0.80 (33%)	−0.85	−1.21 to −0.50	<0.001
	PBM	2.03	0.82	1.21 (59%)			
**Exudate**	Historical control	2.42	1.64	0.78 (32%)	−0.72	−1.07 to −0.38	<0.001
	PBM	2.12	0.91	1.21 (57%)			
**Pain**	Historical control	2.50	1.76	0.74 (30%)	−0.79	−1.21 to −0.38	<0.001
	PBM	2.24	0.97	1.26 (57%)			
**Pruritus**	Historical control	1.62	1.24	0.38 (23%)	−0.59	−1.03 to −0.14	0.011
	PBM	1.26	0.56	0.71 (56%)			

Notes. Values are observed means on a 0–3 scale. Reduction represents the decrease from 24 to 72 h, expressed in points and as a percentage of the 24 h mean. Adjusted differences compare PBM with historical control at 72 h; negative values favor PBM. Model covariates and HC3 inference are described in Section 2.7. Analyses were exploratory, without adjustment for multiple testing. Abbreviations: CI: confidence interval; PBM: photobiomodulation.

## Data Availability

The datasets generated and/or analysed during the current study are available from the corresponding author on reasonable request.

## Supplementary Materials

The following supporting information can be downloaded at: https://www.mdpi.com/article/10.3390/ebj7040050/s1; Table S1: Operational definitions and scoring criteria for the individual domains of the exploratory composite inflammation score; Table S2: Initial local treatment and wound-bed preparation by study group.

## Abbreviations

The following abbreviations are used in this manuscript:

A&E	Accident and emergency
CI	Confidence interval
DACC	Dialkylcarbamoyl chloride
EU	European Union
GDPR	General Data Protection Regulation
GLG	Glucose oxidase, lactoperoxidase, guaiacol
HC	Heteroskedasticity-consistent
IRCCS	Istituto di Ricovero e Cura a Carattere Scientifico
PBM	Photobiomodulation
PHMB	Polyhexamethylene biguanide
SD	Standard deviation
SMD	Standardized mean difference
TBSA	Total body surface area
VSS	Vancouver Scar Scale
WHO	World Health Organization
