# Supplementary material for: Blue-Light Photobiomodulation in Pediatric Burns: Inflammation, Healing, and Early Scar Outcomes in a Retrospective Historical Cohort
Source: Eur Burn J. 2026 Sep 28;7(4):50. doi: 10.3390/ebj7040050 (PMC13692398; doi:10.3390/ebj7040050)
Supplement: Supplementary file 1 [file ebj-07-00050-s001.zip › Supplementary Table S1.pdf]

Supplementary Table S1. Operational definitions and scoring criteria for the individual domains of the exploratory composite inflammation score

Domain	Original assessment	Score 0	Score 1	Score 2	Score 3
**Erythema**	Clinical assessment of erythema surrounding/involving the burn wound	Absent	Mild, localized erythema	Moderate, clearly visible erythema involving a larger proportion of the wound/periwound area	Marked or extensive erythema
**Exudate**	Clinical assessment according to the amount of wound exudate	Absent	Low	Moderate	High
**Pain–FLACC**	FLACC, 0–10 [24]; used in non-verbal children or when reliable self-report was not possible	0	1–3	4–6	7–10
**Pain–FPS-R**	Faces Pain Scale–Revised, 0–10 [25,27]; used when reliable facial self-report was possible	0–2	4	6	8–10
**Pain–NRS**	Numeric Rating Scale, 0–10 [26,28]; used in older children with adequate numerical understanding	0–1	2–5	6–7	8–10
**Pruritus**	Patient self-report; caregiver report and clinical observation when reliable self-report was not possible	Absent	Mild/occasional, without relevant interference	Moderate/recurrent, with observable discomfort or scratching	Severe/persistent, with marked discomfort or frequent scratching

Abbreviations: FLACC, Face, Legs, Activity, Cry, Consolability; FPS-R, Faces Pain Scale–Revised; NRS, Numeric Rating Scale.

Methodological note. Pain was assessed using the original validated instrument appropriate to the child's age, developmental level, and communication abilities. Original pain scores were retained in the clinical record and recoded to a common 0–3 ordinal metric solely to harmonize pain with erythema, exudate, and pruritus for calculation and statistical analysis of the exploratory composite inflammation score. The three pain rows represent alternative instruments: only one recoded pain score contributes to the composite at each assessment. A recoded pain score of 0 denotes the lowest category of the selected instrument and does not invariably indicate absence of pain. The four domains were summed (range 0–12), with higher scores indicating a greater overall burden of local inflammatory signs and symptoms. Recoding did not replace or modify the original validated clinical pain assessment.

Interpretation of recoding. FPS-R categories follow the pediatric acute-pain study by Tsze et al. [27], and NRS categories follow the study by Tsze et al. [28]. FPS-R values are restricted to 0, 2, 4, 6, 8, and 10; 0–2 therefore denotes 0 or 2. The FLACC grouping is an operational recoding adopted for this analysis; reference [24] supports the original instrument, not validation of this transformation. The common metric does not establish equivalence between instruments. Neither the recoding nor the composite has been validated specifically for pediatric burns; the composite is an exploratory measure, not a validated clinical scale. Reference numbers correspond to those in the main manuscript.

References (numbering as in the main manuscript)

[24] Merkel SI, Voepel-Lewis T, Shayevitz JR, Malviya S. The FLACC: a behavioral scale for scoring postoperative pain in young children. Pediatr Nurs. 1997;23(3):293-297.

[25] Hicks CL, von Baeyer CL, Spafford PA, van Korlaar I, Goodenough B. The Faces Pain Scale-Revised: toward a common metric in pediatric pain measurement. Pain. 2001;93(2):173-183. doi:10.1016/S0304-3959(01)00314-1

[26] von Baeyer CL, Spagrud LJ, McCormick JC, Choo E, Neville K, Connelly MA. Three new datasets supporting use of the Numerical Rating Scale (NRS-11) for children's self-reports of pain intensity. Pain. 2009;143(3):223-227. doi:10.1016/j.pain.2009.03.002

[27] Tsze DS, Hirschfeld G, Dayan PS, Bulloch B, von Baeyer CL. Defining No Pain, Mild, Moderate, and Severe Pain Based on the Faces Pain Scale-Revised and Color Analog Scale in Children With Acute Pain. Pediatr Emerg Care. 2018;34(8):537-544. doi:10.1097/PEC.0000000000000791

[28] Tsze DS, Hirschfeld G, Dayan PS. Clinical Interpretation of Self-Reported Pain Scores in Children with Acute Pain. J Pediatr. 2022;240:192-198.e2. doi:10.1016/j.jpeds.2021.08.071
