# Supplementary material for: Blue-Light Photobiomodulation in Pediatric Burns: Inflammation, Healing, and Early Scar Outcomes in a Retrospective Historical Cohort
Source: Eur Burn J. 2026 Sep 28;7(4):50. doi: 10.3390/ebj7040050 (PMC13692398; doi:10.3390/ebj7040050)
Supplement: Supplementary file 1 [file ebj-07-00050-s001.zip › Supplementary Table S2.pdf]

Supplementary Table S2. Initial local treatment and wound-bed preparation by study group.

Variable	Overall (n=84)	Historical Control (n=50)	PBM (n=34)
**Initial local treatment, n (%)**			
Sodium hypochlorite 0.05% antiseptic	13 (15.5)	12 (24.0)	1 (2.9)
Collagenase plus sodium hyaluronate	4 (4.8)	2 (4.0)	2 (5.9)
Silver gelling fiber dressing	23 (27.4)	11 (22.0)	12 (35.3)
Silver alginate dressing	8 (9.5)	6 (12.0)	2 (5.9)
DACC dressing	15 (17.9)	12 (24.0)	3 (8.8)
Hydrogel	17 (20.2)	6 (12.0)	11 (32.4)
Paraffin gauze	4 (4.8)	1 (2.0)	3 (8.8)
**Wound-bed preparation, n (%)**			
Dermabrasion	40 (47.6)	24 (48.0)	16 (47.1)
Enzymatic debridement with NexoBrid	44 (52.4)	26 (52.0)	18 (52.9)
**Wound-bed preparation and grafting, n (%)**			
NexoBrid with grafting	10 (11.9)	8 (16.0)	2 (5.9)
NexoBrid without grafting	34 (40.5)	18 (36.0)	16 (47.1)
Dermabrasion with grafting	22 (26.2)	12 (24.0)	10 (29.4)
Dermabrasion without grafting	18 (21.4)	12 (24.0)	6 (17.6)

Values are presented as n (%), with percentages calculated using the corresponding column totals. Initial local treatment refers to the first treatment recorded in the database and does not describe all products used during subsequent care. One initial treatment category and one wound-bed preparation category were recorded for each patient. DACC: dialkyl-carbamoyl chloride; PBM: photobiomodulation. The four combinations of wound-bed preparation and grafting are mutually exclusive and together account for all 84 patients; grafting could follow either preparation method.
